# Qualitative and Quantitative Assessment of Adenosine Triphosphate Stress Whole-Heart Dynamic Myocardial Perfusion Imaging Using 256-Slice Computed Tomography

**DOI:** 10.1371/journal.pone.0083950

**Published:** 2013-12-23

**Authors:** Akira Kurata, Naoto Kawaguchi, Teruhito Kido, Katsuji Inoue, Jun Suzuki, Akiyoshi Ogimoto, Jun-ichi Funada, Jitsuo Higaki, Masao Miyagawa, Mani Vembar, Teruhito Mochizuki

**Affiliations:** 1 Department of Diagnostic and Therapeutic Radiology, Ehime University Graduate School of Medicine, Ehime, Japan; 2 Department of Integrated Medicine and Informatics, Ehime University Graduate School of Medicine, Ehime, Japan; 3 Department of Cardiology, National Hospital Organization, Ehime National Hospital, Ehime, Japan; 4 CT Clinical Science, Philips Healthcare, Cleveland, Ohio, United States of America; University of Washington School of Medicine, United States of America

## Abstract

**Background:**

The aim of this study was to investigate the correlation of the qualitative transmural extent of hypoperfusion areas (HPA) using stress dynamic whole-heart computed tomography perfusion (CTP) imaging by 256-slice CT with CTP-derived myocardial blood flow (MBF) for the estimation of the severity of coronary artery stenosis.

**Methods and Results:**

Eleven patients underwent adenosine triphosphate (0.16 mg/kg/min, 5 min) stress dynamic CTP by 256-slice CT (coverage: 8 cm, 0.27 s/rotation), and 9 of the 11 patients underwent coronary angiography (CAG). Stress dynamic CTP (whole–heart datasets over 30 consecutive heart beats in systole without spatial and temporal gaps) was acquired with prospective ECG gating (effective radiation dose: 10.4 mSv). The extent of HPAs was visually graded using a 3-point score (normal, subendocardial, transmural). MBF (ml/100g/min) was measured by deconvolution. Differences in MBF (mean ± standard error) according to HPA and CAG results were evaluated. In 27 regions (3 major coronary territories in 9 patients), 11 coronary stenoses (> 50% reduction in diameter) were observed. In 353 myocardial segments, HPA was significantly related to MBF (*P* < 0.05; normal 295 ± 94; subendocardial 186 ± 67; and transmural 80 ± 53). Coronary territory analysis revealed a significant relationship between coronary stenosis severity and MBF (*P* < 0.05; non-significant stenosis [< 50%], 284 ± 97; moderate stenosis [50–70%], 184 ± 74; and severe stenosis [> 70%], 119 ± 69).

**Conclusion:**

The qualitative transmural extent of HPA using stress whole-heart dynamic CTP imaging by 256-slice CT exhibits a good correlation with quantitative CTP-derived MBF and may aid in assessing the hemodynamic significance of coronary artery disease.

## Introduction

Recent technical advancements in multi-detector row computed tomography (MDCT) have enabled coronary CT angiography (coronary CTA) to become a preferred non-invasive technique to assess coronary artery stenosis [1,2] and atherosclerotic plaque [3,4] in patients with coronary artery disease (CAD).

Myocardial perfusion abnormality, which is the first step in the ischemic cascade, has been evaluated with nuclear medicine [5-8], magnetic resonance (MR) imaging [9,10], and echocardiography [11] for risk stratification of myocardial ischemia. Recently, several studies have shown that pharmacological stress myocardial CT perfusion (CTP) imaging can be used to evaluate myocardial ischemia by two methods [12-20]. Single-phase first-pass CTP imaging by retrospective or prospective electrocardiogram (ECG) gated acquisition allows us to evaluate qualitative methods such as CT attenuation based hypoperfusion areas (HPA) [12-15], and dynamic CTP imaging enables us to estimate quantitative parameters such as myocardial blood flow (MBF) [16-19]. Dynamic CTP imaging has the advantage of capturing an entire dynamic series of contrast-enhanced myocardium in comparison with single-phase first-pass CTP imaging. While, no attempt to evaluate HPA using dynamic CTP imaging has been reported previously, recent investigations involved the acquisition of stress dynamic CTP images in alternative positions to cover the heart, causing a temporal gap in the volumetric data [17-19].

A wider MDCT, which has a width covering the entire left ventricular portion, allows us to evaluate whole heart dynamic CTP imaging with neither spatial nor temporal gap. The present study aimed to (1) investigate whether qualitative assessment of HPA in stress dynamic whole-heart consecutive beat CTP imaging by 256-slice MDCT correlated well with CTP-derived MBF, and (2) assess the diagnostic accuracy of the qualitative transmural extent of HPA for detecting coronary artery stenosis in comparison with CTP-derived MBF.

## Materials and Methods

### Study population

This study was approved by Ehime University institutional review board and written informed consent was obtained from all patients. Thirteen patients with CAD (12 men, mean age 61 ± 7 y) who were scheduled for coronary angiography (CAG) were prospectively enrolled and underwent ATP stress dynamic CTP from August 2010 to June 2011. Inclusion criteria were as follows: (1) effort or rest stable angina documented with ST-T change on ECG and (2) asymptomatic patients with at least 3 coronary risk factors of CAD. The exclusion criteria included the following: (1) acute myocardial infarction, (2) unstable angina, (3) chronic atrial fibrillation, (4) chronic kidney disease (serum creatinine, > 1.5 mg/dl), (5) pregnancy, (6) severe left ventricular dysfunction (left ventricular ejection fraction, < 20%), (7) known history of bronchial asthma, (8) symptomatic congestive heart failure, (9) greater than second degree atrio-ventricular block, (10) hyperthyroidism, and (11) known allergic reaction to contrast media.

### ATP-stress dynamic CTP imaging

Patients were ordered to discontinue theophylline and anti-anginal drugs and instructed to avoid caffeine 24 h before the ATP-stress test. No beta-blocker was orally or intravenously administered prior to the present study in order to reduce heart rate.

**Figure 1:**
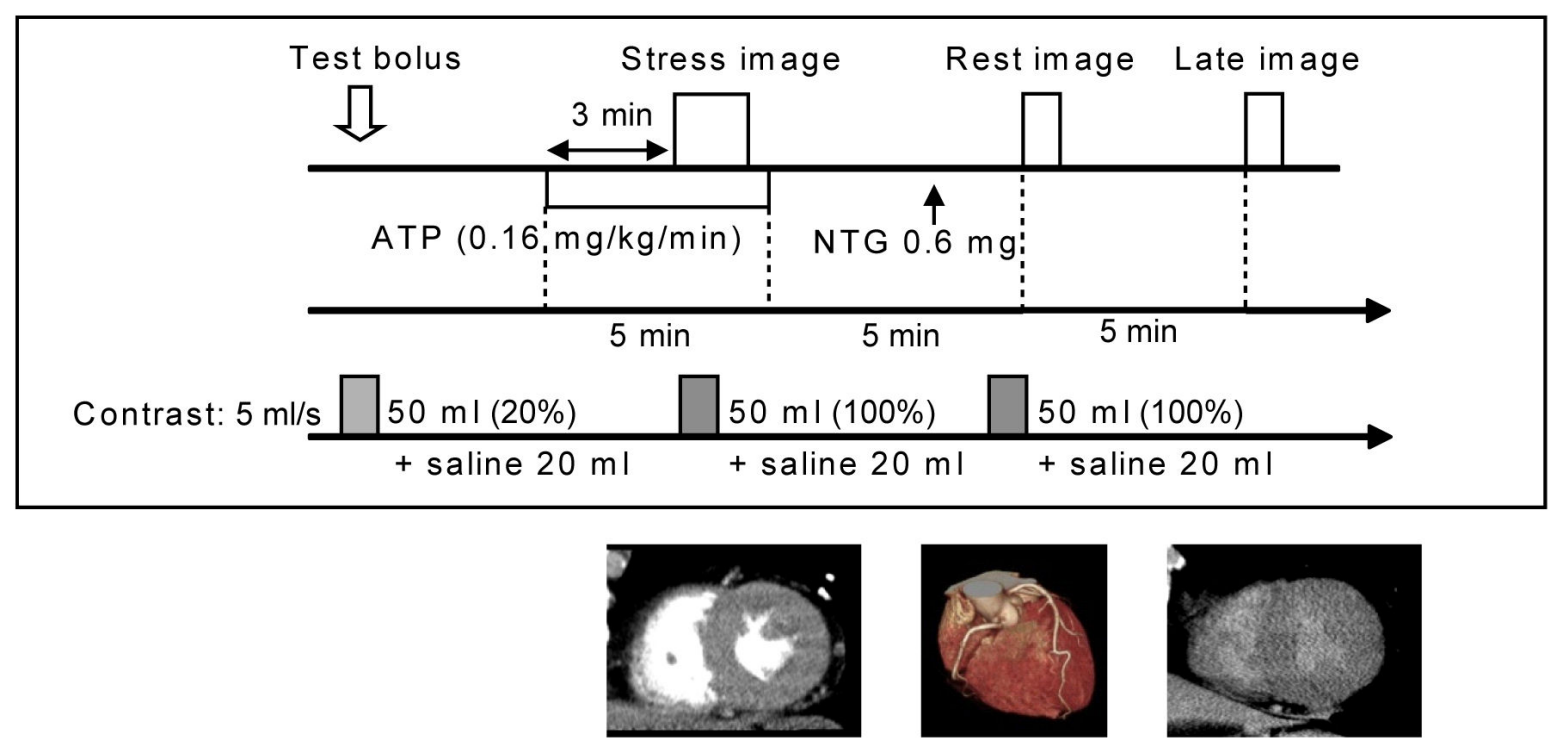
ATP–stress dynamic CT perfusion imaging protocol.

We used a 256-slice MDCT (Brilliance iCT, Philips Healthcare, Cleveland OH, USA) with 8 cm of cranio-caudal coverage and gantry rotation time of 0.27 s. A prospective ECG-gated dynamic acquisition mode was installed for this study (work in progress, Philips Healthcare, Cleveland OH, USA). An automatic dual injector (Stellant DualFlow; Nihon Medrad KK, Osaka, Japan) was used for the administration of contrast medium. The scanning protocol consisted of collecting pharmacological stress dynamic perfusion images for myocardial ischemia, rest images for coronary CTA, and late images for myocardial infarction (Figure 1, Table 1). First, a test bolus scan using an automatically 20%-diluted solution (5 ml/s for 10 s) of contrast medium (Iopamidol 370 mg iodine/ml; Bayer Yakuhin, LTD, Osaka, Japan) and 0.9% sodium chloride (Otsuka normal saline; Otsuka Pharmaceutical Co., Ltd, Tokyo, Japan) followed by a saline chaser (5 ml/s for 4 s) was performed at the proximal part of the ascending aorta. The scan delay was determined as 6 s before the time to peak enhancement of the ascending aorta for collection of dynamic CTP data; this also allowed us to establish a baseline prior to the arrival of contrast in the aorta. Second, pharmacological stress was induced with ATP loading (ATP 20mg; Daiichi Sankyo Inc, Tokyo, Japan 0.16 mg/kg/min, 5 min) as described by Miyagawa et al. [6]. Three minutes after ATP loading, patients were instructed to hold their breath in the expiration position to shorten the craniocaudal length of the heart, and CTP images were obtained using the prospective ECG-gated dynamic acquisition mode triggered at a phase of 40% RR interval (systolic phase) during the administration of contrast medium (5 ml/s for 10 s) followed by a saline chaser (5 ml/s for 4 s). Third, coronary CTA images were obtained with prospective ECG gating at a phase of 75% RR interval (mid-diastolic phase) under a single breath-hold in the inspiration position. Finally, late CT images were obtained 5 min after coronary CTA with prospective ECG gating at a phase of 40% RR interval (systolic phase) under a single breath-hold in the inspiration position without additional contrast medium. Patients’ standard ECG, vital signs, and general condition were continuously monitored during the stress protocol by a cardiologist and a radiologist. The estimated radiation doses were 10.4, 4.3, and 3.2 mSv, respectively, for the CTP, coronary CTA and late CT scans.

**Table 1:** Scan parameters of the ATP-stress dynamic CT perfusion imaging.

	Stress image	Rest image	Late image
Scan mode	Dynamic scan	Axial scan	Axial scan
ECG triggering	Prospective	Prospective	Prospective
Cardiac phase	RR 40%	RR 75%	RR 40%
Phase tolerance (%)	0	5	0
Tube voltage (kV)	100	100	100
Tube current (mAs/rotation)	80	210	210
Rotation speed (s/rotation)	0.27	0.27	0.27
Detector collimation (mm)	64 × 1.25	128 × 0.625	128 × 0.625
Coverage (cm)	8	Whole heart	Whole heart
Scan time	30 consecutive beats	3-5 s	3-5 s
Reconstruction	Full reconstruction	Half reconstruction	Half reconstruction
Estimated dose (mSv)	10.4	4.3	3.1

Radiation dose is estimated from cardiac phantom study.

### Reconstruction of stress dynamic CTP imaging

Trans-axial images from the stress dynamic CTP scans were reconstructed using a 360° reconstruction algorithm, and the noise was reduced using a spatio-temporal diffusion filter (work in progress, Philips Healthcare, Cleveland OH, USA). A dynamic series of contiguous short-axis views without spatial gaps from the apex to the level of the left ventricular outflow tract (mean number of slices, 20; range, 16–27) were reconstructed with a slice thickness of 3 mm, and displayed in maximum intensity projection mode with a window level of 100 Hounsfield Units (HU) and a window width of 200 HU using a dedicated workstation (Extended Brilliance Workspace, Philips Healthcare, Cleveland OH, USA).

### Qualitative assessment

Qualitative assessment was performed by 2 experienced radiologists, who were blinded to clinical or diagnostic imaging findings and had experience with over 50 cases of stress CTP imaging. Using whole-heart dataset where 30 consecutive heartbeats were obtained, CTP patterns were visually classified into 3 groups based on HPA grade: (1) transmural HPA, including more than 50% of wall thickness; (2) subendocardial HPA, including less than 50 % of wall thickness; and (3) normal, no HPA.

The HPA extent was evaluated for each left ventricular segment based on a standard 16-segment model excluding the apical region, and was classified into 3 major coronary vessels and their corresponding myocardial territories: the left anterior descending artery (LAD segments 1, 2, 7, 8, 13, 14); the left circumflex artery (LCX segments 5, 6, 11, 12, 16); and the right coronary artery (RCA segments 3, 4, 9, 10, 15) [21].

### Quantitative analysis

Quantitative assessment was also independently performed by 2 other experienced radiologists.

The same datasets were analyzed with a commercially available program using a deconvolution method (CT Perfusion 4 in Advantage Windows 4.6, GE Healthcare, Milwaukee, WI, USA). Briefly, the region of interest (ROI) set in the left ventricular cavity was selected as the input function for the perfusion calculation. The time-density curve of the left ventricular myocardium was constructed from 30 images and model-based deconvolution analysis was applied to the input function. On the basis of the central volume principle, MBF was calculated from the formula: MBF = myocardial blood volume/mean transit time [22–24].

The ROI was set in the middle layer of the left ventricular myocardium (2-3 ROIs per segment, size of ROI: 15-22 mm^2^, all cardiac short-axis slices), independently from the qualitative assessment of HPA. We defined the mean MBF of all ROIs in each segment as the segmental MBF. While analyzing the time-density curves and dynamic cine CTP images, ROIs in areas affected by motion artifacts from cardiac motion or irregular heartbeats that resulted in excessive noise spikes in the corresponding time-density curve were excluded. When no evaluable ROI was present in a given myocardial segment, the segment was also excluded.

### Coronary CT angiography

Axial images with a slice thickness of 0.8 mm and a section interval of 0.4 mm were reconstructed using a medium cardiac kernel (XCB). Datasets were analyzed on a dedicated CT workstation (Extended Brilliance Workspace, Philips Healthcare, Cleveland, OH, USA). Coronary arteries were assessed based on the American Heart Association 15-segment model [25]. The presence of coronary stenosis was assessed with lesions defined as follows: normal – absence of plaque with no luminal stenosis; minimal – plaque with < 25% stenosis; mild – 25-49% stenosis; moderate: 50-69% stenosis; severe: 70-99% stenosis; and total occlusion. Coronary stenosis ≥ 50% was defined as significant. Coronary segments that were non-assessable because of extensive calcium and the presence of motion artifacts were assumed to be having significant disease. When multiple lesions were present, the corresponding segment was classified by the worst lesion.

### Conventional CAG

Standard CAG was performed using 5-Fr catheters. All angiograms were documented on CD-ROM and quantitatively evaluated by 2 experienced cardiologists (A.O. and J.H.) who were unaware of other investigation parameters.

The coronary artery was assessed per segment based on the 15-segment model [25]. Using commercially available software (QCA-CMS system version 3.0, MEDIS, Leiden, The Netherlands), the severity of coronary artery stenosis was classified as follows: severe stenosis (> 70% reduction in diameter), moderate stenosis (50-70%), and non-significant stenosis (

< 50%). When a given segment included multiple lesions, the segment was classified by the worst lesion. When a given coronary artery included multiple abnormal segments, the coronary artery was classified by the worst segment.

In the present study, the impact of the worst segment in a given coronary artery was applied to all the myocardial segments associated with the artery.

### Statistical analysis

Data are presented as the mean value ± standard error (SE) as appropriate according to distribution. First, the interobserver variability for qualitative assessment of stress CTP images was calculated with the Cohen κ-value. Second, concerning the transmural extent of HPA based on the 16-segment model, the differences in MBF values across all groups were analyzed using 1-factor factorial analysis of variance, and the differences in MBF values between any 2 of the 3 groups were analyzed using a Bonferroni test. Finally, concerning the severity of coronary artery stenosis based on 3 major coronary vessels and territories, the differences in MBF values across all groups were analyzed using 1-factor factorial analysis of variance, and the differences in MBF values between any 2 of the 3 groups were analyzed using a Bonferroni test.

We determined the MBF cut-off values for detecting transmural HPA (versus subendocardial HPA and non-HPA) and non-HPA (versus subendocardial and transmural HPA) for each by maximizing the area under the receiver operating characteristic (ROC) curves, and used them to determine sensitivity, specificity, positive, and negative predictive values (PPV and NPV) of the HPA-based MBF cut-off values for detecting obstructive CAD, which was defined by CAG at 2 thresholds ( > 70% or > 50% stenosis severity). Additionally, these results were compared to those estimated by the cut-off values for MBF alone.

Moreover, we calculated sensitivity, specificity, PPV and NPV of coronary CTA alone and also for the combined assessment of coronary CTA and MBF for detecting significant stenosis (> 50% stenosis severity) defined by CAG. When a given coronary vessel territory from coronary CTA indicated coronary stenosis with the corresponding myocardial segments having MBF lower than the cut-off value, the coronary vessel territory was judged as positive for the combined assessment of coronary CTA and MBF.

All statistical analyses were performed with SPSS Version 21 (SPSS Institute Inc, Chicago, IL, USA). The threshold of significance was set at *P* < 0.05.

## Results

Patient characteristics are shown in Table 2. The 10-year CAD death pre-test probabilities were 0.5-1% (n = 2), 1-2% (n = 4), 2-5% (n = 4), and > 10% (n = 1) according to a risk assessment based on a 19-year follow-up study of a Japanese representative population (NIPPON DATA 80) based on sex, age, systolic blood pressure, smoking, and serum total cholesterol and glucose levels [26]. 

**Table 2:** Patient Characteristics.

Age, y	61 ± 8
Male, n	12
Weight, kg	67 ± 11
Hypertension, n	10
Diabetes, n	9
Smoking history, n	6
Family history of CAD, n	5
Symptom, n	
Effort angina	6
Rest angina	6
Asymptomatic	1

CAD = coronary artery disease

**Figure 2:**
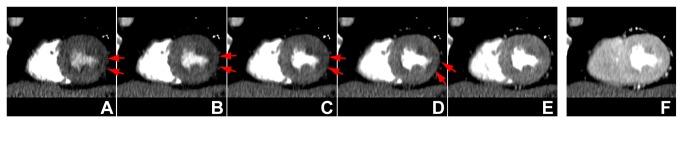
ECG gated stress dynamic CT perfusion imaging over 30consecutive heart beats of a 70 year-old man with symptomatic coronary artery disease. Cardiac short-axis views of the early 5 phases (A, B, C, D, E) are shown, and the phase with peak enhancement (F) demonstrates transient subendocardial hypoperfusion areas (red arrows) in the lateral wall of the left ventricular myocardium. Data acquisition over consecutive heartbeats allowed us to perform a robust qualitative assessment of the myocardium without missing the optimal phase from the true volumetric data without temporal gaps.

All 13 patients underwent the ATP-stress dynamic CTP study without major complication to warrant termination of the ATP infusion. The mean heart rate (beat/min, mean ± standard deviation) in the steady state before the ATP infusion, stress, and rest (coronary) imaging were 60 ± 7 bpm, 74 ± 11 bpm, and 64 ± 5 bpm, respectively. In 2 of 13 patients, whole-heart dynamic stress CTP data could not be acquired, because their heart position was slightly deviated from the coverage of the 256-slice MDCT (less than 2 cm and only in the apical inferior segment); in the remaining 11 patients, whole-heart dynamic series of contiguous short-axis views were successfully acquired and evaluated with neither spatial nor temporal gaps (Figures 1, 2). Coronary CTA was successfully performed in all 13 patients. Two patients refused CAG because of the absence of significant stenosis on coronary CTA and/or a history of myocardial infarction (post-infarction fatty tissue). Because stress dynamic CTP imaging had insufficient coverage (truncation issue) in 2 patients, and no coronary stenosis on coronary CTA was found in 2 other patients, 9 patients ultimately underwent CAG and were included in the analysis.

Concerning the reproducibility of the qualitative transmural extent of HPA, concordance of the observers was 0.81 in 5 selected patients (80 ROIs), and we concluded that this reliability was satisfactory (κ > 0.70). 

Among 9 patients, CAG depicted 4 coronary territories with severe coronary stenosis (> 70%), 7 coronary territories with moderate coronary stenosis (50–70%) and 16 coronary territories without significant coronary stenosis. Coronary CTA detected 6 severe coronary stenoses (> 70%) and 9 moderate coronary stenoses (50 - 70%). One coronary segment could not be evaluated because of extensive calcification. Thus a total of 16 coronary vessels were assumed to have clinically significant CAD.

### Transmural extent and MBF

On the basis of the 16-segment model, 3 cardiac short-axis slices per segment were evaluated in 6 patients (3 × 16 × 6 = 288 ROIs), and 2 cardiac short-axis slices per segment were evaluated in 3 patients (2 × 16 × 3 = 96 ROIs). A total of 384 ROIs were estimated in the study. Thirty-one ROIs could be evaluated by qualitative assessment but could not be assessed quantitatively by MBF because of motion artifacts, and these ROIs were excluded from the analysis. Finally, a total of 353 ROIs were included for the assessment of the transmural extent of low attenuation area and MBF. We observed 139 ROIs without HPA (normal), 154 ROIs with subendocardial HPA, and 60 ROIs with transmural HPA.

**Figure 3:**
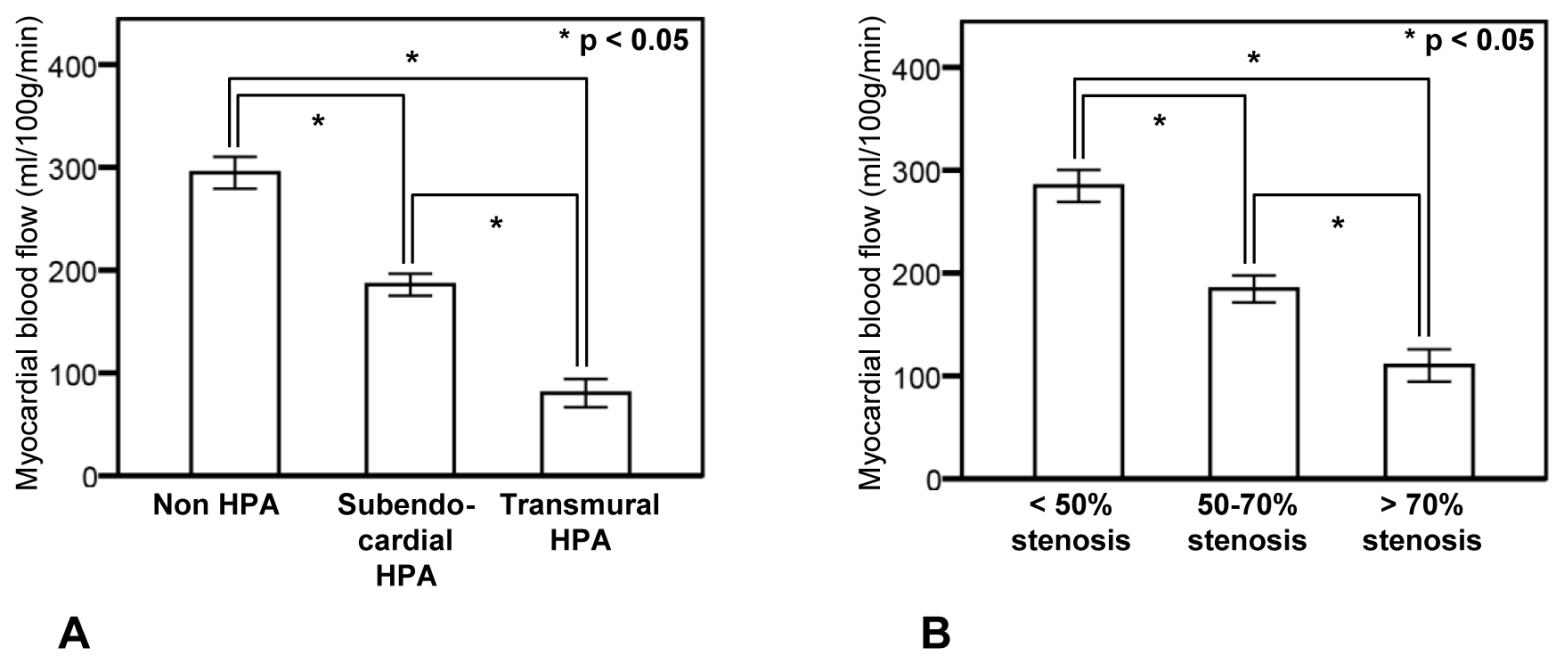
Qualitative and quantitative assessment of stress dynamic CT perfusion imaging and coronary artery stenosis. A: Relationship between the transmural extent of hypoperfusion area and myocardial blood flow. B: Relationship between coronary artery stenosis and myocardial blood flow. Boxes with error bars indicate mean values ± standard error. Myocardial blood flow was significantly decreased as the severity of the transmural extent of the hypoperfusion area (HPA) increased from non-HPA to transmural HPA (*P* < 0.05). Myocardial blood flow was significantly decreased as the severity of coronary stenosis increased (*P* < 0.05).

Mean MBF values for transmural HPA, subendocardial HPA and no HPA were 80 ± 7 ml/100 g/min, 186 ± 5 ml/100 g/min, and 295 ± 8 ml/100 g/min, respectively. MBF values significantly differed across the 3 groups (*P* < 0.05). Post-hoc analysis revealed that MBF was significantly lower in the subendocardial and transmural HPA groups than in the non–HPA group (*P* < 0.05; Figure 3A), and MBF was significantly lower in the transmural HPA group than in the subendocardial HPA group (*P* < 0.05; Figure 3A).

### Coronary artery stenosis and MBF

On the basis of the presence and location of coronary stenoses, the number of ROIs in the myocardial segments corresponding to severe coronary stenoses (> 70%), moderate coronary stenoses (50-70%) and non-significant coronary stenoses (< 50%) were 77, 126, and 150, respectively.

Mean MBF values in myocardial segments without significant stenosis (< 50%) and with moderate (50-70%) and severe (> 70%) stenosis were 284 ± 8 ml/100 g/min, 184 ± 7 ml/100 g/min, and 110 ± 8 ml/100 g/min, respectively. MBF values significantly differed across the 3 groups (*P* < 0.05; Figure 3B). Post-hoc analysis revealed that MBF was significantly lower in the moderate and severe stenosis groups than in the non-significant stenosis group, and MBF of the severe stenosis group was significantly lower than that of moderate stenosis group (*P* < 0.05; Figure 3B).

### Diagnostic accuracy for detecting coronary artery stenosis

Cut-off MBF values for detecting transmural HPA and excluding non-HPA were 138.5 ml/100 g/min (area under the curve [AUC] 0.957; 95% confidence interval [CI] 0.933–0.981) and 193.5 ml/100 g/min (AUC 0.902; 95% CI 0.871–0.934). Cut-off MBF values for detecting obstructive CAD at 2 thresholds of > 70% and > 50% stenosis severity were 174.5 ml/100 g/min (AUC 0.869; 95% CI: 0.825–0.913) and 184.5 ml/100 g/min (AUC 0.871; 95% CI 0.834–0.908), respectively. Diagnostic accuracy of the HPA-based and ROC-based MBF cut-off values are shown in Table 3 and Table 4.

**Table 3:** Diagnostic accuracy for detecting coronary stenosis with > 70% lumen diameter reduction.

	Sensitivity	Specificity	PPV	NPV
HPA-based MBF cut-off (138.5 ml/100 g/min)	47/77, 61% (49.2-71.7%)	245/276, 89% (84.3-92.1%)	47/78, 60% (48.5-71%)	245/275, 89% (86.4-92.4%)
ROC-based MBF cut-off (174.5 ml/100g/min)	68/77, 88% (78.5-94.2%)	188/276, 68% (62.2-73.5%)	68/156, 44% (35.7-51.8%)	188/197, 95% (91.2-97.7%)

HPA-based MBF cut-off (138.5 ml/100g/min): myocardial blood flow cut-off value for detecting the quantitative transmural extent of hypoperfusion area (HPA). ROC-based MBF cut-off (174.5 ml/100g/min): myocardial blood flow cut-off value for detecting severe coronary artery stenosis (> 70%). PPV and NPV: positive and negative predictive values. Data are expressed as n/N, %, 95% confidence interval.

**Table 4:** Diagnostic accuracy for detecting coronary stenosis with > 50% lumen diameter reduction.

	Sensitivity	Specificity	PPV	NPV
HPA-based MBF cut-off (193.5 ml/100 g/min)	160/203, 79% (72.4-84.1%)	124/150, 83% (75.4-88.2%)	160/186, 86% (80.0-90.5%)	124/167, 74% (66.8-80.6%)
ROC-based MBF cut-off (184.5 ml/100g/min)	152/203, 75% (68.2-80.6%)	131/150, 87.3% (80.7-92.0%)	152/171, 89% (83.0-93.0%)	131/182, 72% (64.8-78.2%)

HPA-based cut-off MBF (193.5 ml/100g/min): myocardial blood flow cut-off value for excluding non-HPA. ROC-based MBF cut-off (184.5 ml/100g/min): myocardial blood flow cut-off value for detecting significant coronary artery stenosis (> 50%). PPV and NPV: positive and negative predictive values. Data are expressed as n/N, %, 95% confidence interval.

### Incremental value of MBF on coronary CTA for detecting coronary artery stenosis

The results of the diagnostic accuracy of coronary CTA alone and combined assessment of coronary CTA and MBF (cut-off value: 184.5 ml/100 g/min) are shown in Table 5. Combined with MBF, specificity and PPV improved from 11/16 (68.8%, 95%CI 41.4-88.8%) and 11/16(68.8%, 95%CI 41.4-88.8%) to 11/14 (78.6%, 95%CI 49.2-95.1%) and 13/16 (81.2%, 95%CI 54.6-95.7%), respectively. Out of the 5 coronary vessels assessed by coronary CTA that were false positive, two were corrected by a combined assessment of coronary CTA and MBF. Only one myocardial territory corresponding to the non-assessable coronary vessel (due to extensive calcification) included some segments with MBF lower than the cut-off value of 184.5 ml/100 g/min; however, there was no added benefit of the incremental value of MBF with coronary CTA for this particular vessel, because CAG revealed non-significant stenosis.

**Table 5:** Diagnostic accuracy for detecting coronary stenosis with > 50% lumen diameter reduction.

	Sensitivity	Specificity	PPV	NPV
Coronary CTA alone	11/11, 100% (71.3-100%)	11/16, 68.8% (41.4-88.8%)	11/16, 68.8% (41.4-88.8%)	11/11, 100% (71.3-100%)
Coronary CTA + MBF cut-off (184.5 ml/100g/min)	13/13, 100% (75.1-100%)	11/14, 78.6% (49.2-95.1%)	13/16, 81.2% (54.6-95.7%)	11/11, 100% (71.3-100%)

Coronary CTA + MBF cut-off (184.5 ml/100g/min): combined assessment of coronary computed tomography angiography and the receiver operating characteristic curve based myocardial blood flow cut-off value for detecting significant coronary artery stenosis (> 50%). PPV and NPV: positive and negative predictive values. Data are expressed as n/N, %, 95% confidence interval.

## Discussion

Our present study investigated the feasibility of qualitative assessment of stress dynamic CTP imaging by 256-slice MDCT to evaluate myocardial ischemia in patients with CAD. We demonstrated that 1) 256-slice MDCT allowed whole-heart dynamic CTP imaging with neither spatial nor temporal gap, and 2) there was a good correlation between the transmural extent of HPA and CTP-derived MBF for the estimation of coronary artery stenosis severity.

### Whole-heart dynamic CTP imaging

With 8-cm coverage along the z-axis, the 256-slice MDCT scanner was able to cover the whole heart in the expiratory breath-hold position and systolic phase for most of our participants, except for initial technical errors in positioning of the heart. Dynamic CTP imaging of the whole heart allowed us to acquire images without spatial and temporal gaps in comparison with dynamic myocardial perfusion using MR [9,10,27] and recently published dynamic CT perfusion imaging methods with alternating table positions [17-19]. Meanwhile, ROI registration failures when measuring MBF were seen in 31 of 353 ROIs (8.8%) resulting from motion artifacts, and these ROIs were excluded from the analysis.

### Qualitative and quantitative assessment of stress dynamic CTP imaging

**Figure 4:**
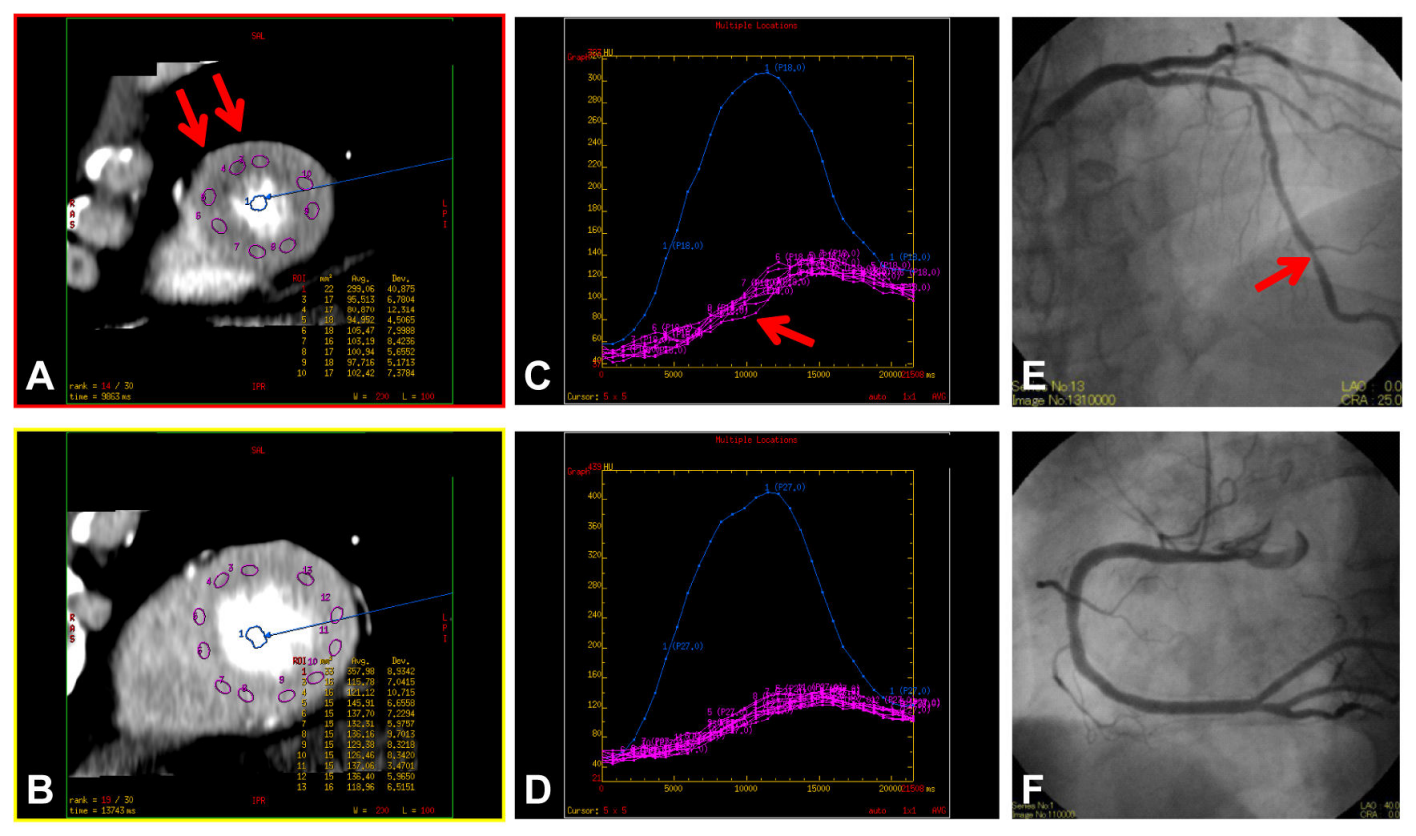
Qualitative and quantitative assessment of myocardial perfusion. An asymptomatic patient in his 60’s with coronary artery disease. Figures 4A and 4B show cardiac short-axis views at the apical and the mid-ventricular level, respectively. The corresponding time attenuation curves within the regions of interest at the 2 levels (C and D, respectively) show a slight delay in contrast enhancement (red arrow, C) in subendocardial HPA in the apical region (red arrow, A), indicating lower myocardial blood flow. Catheter coronary angiography reveals moderate (50–70%) stenosis in the distal portion of the left anterior descending artery (red arrow, E) and no stenosis in the right coronary artery (F).

**Figure 5:**
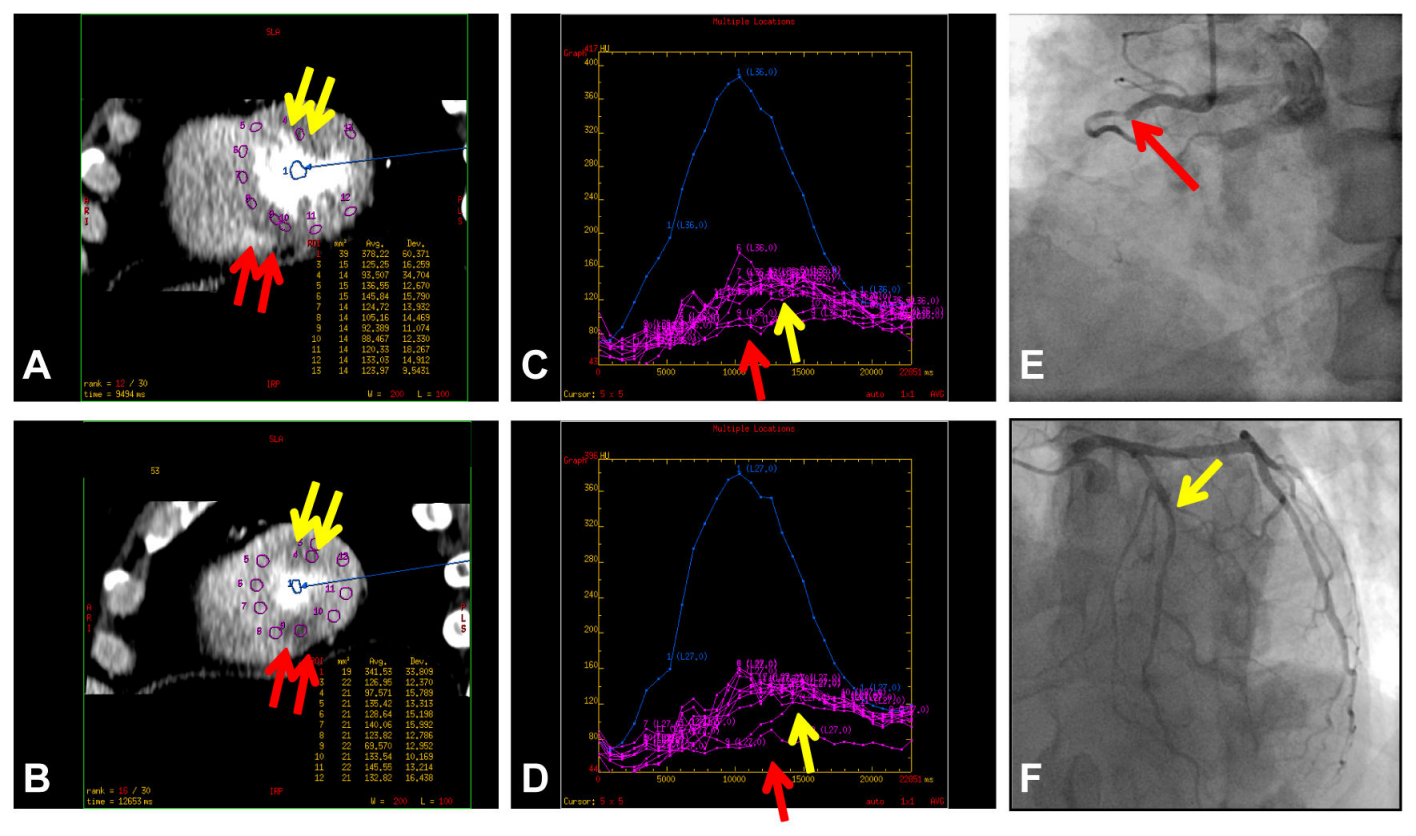
A patient in his 50’s with effort angina. (A and B) Short-axis images of stress dynamic CTP imaging show subendocardial HPA (yellow arrow) in the anterior segments and transmural HPA in the inferoseptal segments (red arrow). (C and D) The corresponding time attenuation curves of them reveal late (yellow arrow) and low (red arrow) contrast enhancement patterns and low myocardial blood flow in the HPAs. (E and F) Catheter coronary angiography reveals severe (90%) stenosis at the second diagonal branch (yellow arrow, F) and total occlusion in the proximal portion of the right coronary artery with collateral artery via the left circumflex artery (red arrow, E).

Because of the high gantry rotation speed, precise prospective ECG gated data acquisition over consecutive heart beats, full 360° reconstruction of the data [28], and dedicated diffusion filter to reduce noise, the quality of the image reconstructions was sufficient for qualitative assessment (Figures 2, 4A, 4B, 5A, and 5B), and robust time attenuation curves of myocardium could be generated for quantitative assessment (Figures 4C, 4D, 5C, and 5D). 

Our data showed that qualitative assessment of HPA was comparable to the ROC-based MBF cut-off values for detecting moderate and severe coronary artery stenoses. These results may indicate that qualitative transmural extent of HPA has the potential to provide an estimate of MBF and the severity of coronary artery stenosis without time-consuming procedures for calculating MBF using a dedicated workstation in clinical practice, while also avoiding motion-related artifacts in MBF calculations.

### Comprehensive assessment of CAD by stress dynamic CTP imaging

The MBF is an absolute value but is often influenced not only by coronary artery stenosis but also coronary risk factors and other cardiac diseases. Even in patients with diabetic microangiopathy, the decrease of MBF in stress dynamic CTP imaging may not always suggest obstructive CAD; additionally, other commonly used measurements such as the ratio of MBF in ischemic areas/MBF in hyperemic area or stress MBF/rest MBF may not be reliable indicators of the presence of disease. Combined assessment of coronary CTA and myocardial ischemia (stress CTP imaging) is useful to assess CAD although assessment of pre-test probability and selection of patients by low-dose coronary CTA before stress CTP imaging should be further investigated. In patients with known or unknown history of myocardial infarction [29,30], assessment of myocardial infarction and residual myocardium is important to predict the therapeutic efficacy of coronary revascularization therapy [9,10]. Our study also indicated a possibility of incremental value of MBF with coronary CTA for detecting coronary artery stenosis. Combined assessment of coronary CTA and MBF could make it feasible to assess the significance of intermediate stenosis [29] especially when some vessels and segments are non-assessable [12].

The present protocol of our study using 256-slice MDCT shows promise for comprehensive assessment of CAD within a single examination that includes stress dynamic CTP (qualitative and quantitative assessment), coronary CTA, and late iodine-enhanced imaging [30].

### Study limitations

The number of patients included in this study was small, and non-randomized patients with a higher prevalence of CAD were included. Culprit coronary stenoses and related myocardial segmentation were not anatomically classified. This study was performed under the assumption that both proximal and distal lesions were classified in the same coronary stenotic artery territory. Classification of myocardial segments and establishing their relationships to the culprit coronary lesions should be objectively performed in further studies. This will allow more precise and effective diagnosis (and analysis) per coronary vessel (territory) and patient. Dynamic CTP imaging allows for assessing not only MBF, but also myocardial blood volume, mean transit time, etc. The clinical significance of these quantitative parameters should be explored in further studies. The coverage of the 256-slice MDCT may be slightly small to encompass the entire heart in some candidates. A motion correction algorithm for measuring quantitative MBF should be given due consideration.

Our findings need to be further validated with other well-established modalities, such as nuclear medicine, MR, and fractional flow reserve measurement using an intra-coronary pressure wire. Estimation of MBF in the present study was performed with commercially available software using a deconvolution method, which also requires further verification. Late-enhancement CT to identify myocardial infarction suffers from lower contrast-to-noise ratio (CNR) in comparison with MR late-enhancement imaging [31]. Radiation dose remains a concern for the assessment of myocardial ischemia using stress dynamic CTP. The use of iterative reconstruction algorithms and low-tube-voltage scanning techniques could help reduce the total radiation dose of the exam without decreasing image quality in stress perfusion and late-enhancement imaging [32].

## Conclusion

Stress whole-heart dynamic CTP imaging without spatial and temporal gaps is feasible with 256-slice CT. The qualitative transmural extent of HPA exhibits a good correlation with quantitative CTP-derived MBF and may aid in assessing the hemodynamic significance of coronary artery disease.
